# Frequent amplification of *AIB1*, a critical oncogene modulating major signaling pathways, is associated with poor survival in gastric cancer

**DOI:** 10.18632/oncotarget.3852

**Published:** 2015-04-29

**Authors:** Jing Shi, Wei Liu, Fang Sui, Rong Lu, Qingyuan He, Qi Yang, Hongjun Lv, Bingyin Shi, Peng Hou

**Affiliations:** ^1^ Department of Endocrinology, The First Affiliated Hospital of Xi'an Jiaotong University School of Medicine, Xi'an 710061, The People's Republic of China; ^2^ Department of Oncology, The First Affiliated Hospital of Xi'an Jiaotong University School of Medicine, Xi'an 710061, The People's Republic of China

**Keywords:** gastric cancer, AIB1, genomic amplification, poor prognosis, signaling pathways

## Abstract

Amplified in breast cancer 1 (AIB1) is a member of p160 steroid receptor coactivator (SRC) family that mediates the transcriptional activities of nuclear receptors and other transcription factors. It acts as a major oncogene in diverse cancers, whereas biological function of AIB1 in gastric cancer remains largely unclear. This study was designed to explore the role of AIB1 in gastric tumorigenesis and its potential as a useful prognostic marker and therapeutic target in this cancer. Our data demonstrated that *AIB1* was significantly up-regulated in gastric cancer tissues as compared with control subjects. Moreover, *AIB1* amplification was found in 47 of 133 (35.3%) gastric cancer cases, but not in control subjects. *AIB1* amplification was positively associated with its protein expression, and was significantly correlated with poor patient survival. AIB1 knockdown in gastric cancer cells dramatically inhibited cell proliferation, invasiveness and tumorigenic potential in nude mice, and induced cell cycle arrest and apoptosis. Mechanically, AIB1 promotes gastric cancer cell proliferation, survival and invasiveness through modulating major signaling pathways such as ErbB and Wnt/β-catenin pathways. Collectively, these findings suggest that AIB1 plays an important role in the pathogenesis of gastric cancer and represents a potential prognostic marker and therapeutic target for this cancer.

## INTRODUCTION

Gastric cancer is one of the most common malignancies and the mortality remains the second leading cause for cancer-related deaths worldwide [1]. Gastric cancer is a heterogeneous disease in terms of histology, anatomy, and epidemiology, although recent diagnostic and therapeutic strategies have gradually advanced, gastric cancer is usually not diagnosed until an advanced stage and the 5-year survival rates are still quite low [2, 3]. Thus, early detection of gastric cancer is extremely important for good patient outcomes and identification of prognostic and predictive biomarkers is critical to predict and guide clinical treatment for this disease.

Gastric cancer is a complex, multistep process involving deregulation of genetic and epigenetic alterations. Genetic alterations, such as gene amplification, gene mutations [4] and polymorphisms [5, 6] are associated with gastric cancer. Gene amplification is one of the most frequent genomic alterations found in human cancers [7, 8], including gastric cancer [9, 10]. Increased gene dosage by this genetic event is a common mechanism for oncogene overexpression during tumorigenesis. Amplified in breast cancer 1 (AIB1) (also known as SRC-3, ACTR, RAC-3, TRAM-1 and p/CIP), is a member of the p160 steroid receptor coactivator (SRC) family [11], which interacts with nuclear receptors (NR) such as estrogen receptor (ER) and androgen receptor (AR), as well as other transcription factors such as E2F1 [12], activator protein-1 (AP-1) [13], nuclear factor-kB (NF-kB) [14] and PEA3 [15] to enhance their effects on target gene transcription. AIB1 has been broadly investigated in hormone-dependent cancers such as prostate cancer [16], ovarian cancer [17] and uterine endometrial cancers [18] since it was initially discovered to be amplified and overexpressed in breast cancer in 1997 [19]. In the past years, a growing body of evidence has shown that *AIB1* is also overexpressed or amplified in several hormone-independent cancers such as hepatocellular carcinoma [20], esophageal squamous cell carcinoma [21], colorectal carcinoma [22], pancreatic adenocarcinoma [23] and cholangiocarcinoma [24]. In addition, the transgenic and knockout mouse models further supported the oncogenic function of AIB1 in tumorigenesis [25, 26].

Although a previous study showed that *AIB1* amplification was observed in 7% and overexpression in 40% primary gastric cancers [27], the exact role of AIB1 in gastric tumorigenesis remains totally unknown. In this study, we found frequent *AIB1* amplification and overexpression in a cohort of gastric cancers, and demonstrated that genomic amplification was one of the major mechanisms for *AIB1* overexpression in gastric cancer. In addition, our data revealed a close association of *AIB1* amplification with poor survival of gastric cancer patients. AIB1 down-regulation significantly reduced *in vitro* and *in vivo* oncogenic potential of gastric cancer cells through modulating major signaling pathways.

## RESULTS

### Frequent overexpression and amplification of *AIB1* in gastric cancer

**Figure 1:**
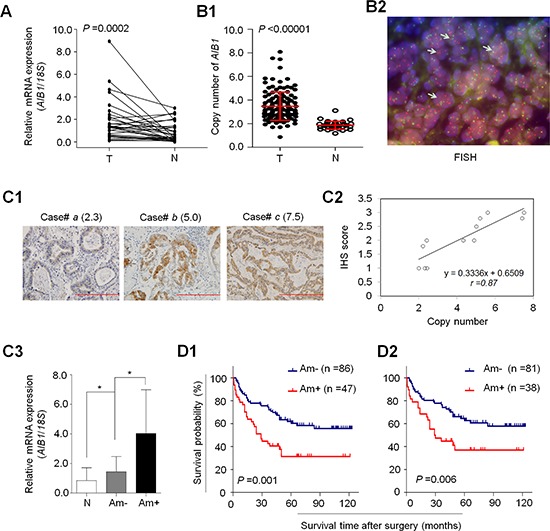
Overexpression and amplification of *AIB1* in gastric cancer **A.** *AIB1* mRNA expression was significantly up-regulated in primary gastric cancers (T) as compared with matched normal gastric tissues (N) as determined by qRT-PCR assay (*n* = 30). *AIB1* expression was normalized with *18S* rRNA levels. **B1.** Real-time quantitative PCR was performed to analyze *AIB1* copy number in a cohort of gastric cancers (T) and control subjects (N). Horizontal lines indicate mean ± SD. **B2.** Bicolor FISH analysis demonstrates *AIB1* amplification (red signals) in primary gastric cancer tissues by using AIB1 DNA probe, and reference centromeric probe on chromosome 20 (CEN20) was shown in green. Arrows indicate the cells with *AIB1* amplification. **C1.** Increasing extent of specific staining (brown color) was associated with increasing *AIB1* copy number (number inside brackets). Shown are representative cases of immunohistostainging on gastric cancer histologic slides using anti-AIB1 antibody. Linear regression analysis was performed to assess the association of AIB1 immunohistostaining score with *AIB1* copy number on 12 randomly selected gastric cancer cases (**C2.**, *r* = 0.87). **C3.** *AIB1* mRNA expression was evaluated by using qRT-PCR assay in primary gastric cancer (*n* = 30) grouping with *AIB1* amplification and matched normal tissues (N). *P < 0.05.*18S* rRNA was used as a normalized control. Data were presented as mean ± SE. Kaplan–Meier survival curves were used to assess the survival of primary gastric cancer patients **D1.** The patients with *AIB1* amplification (Am+) had significantly shorter survival times than the patients without *AIB1* amplification (Am-). When the patients with residual cancers were excluded, the patients with *AIB1* amplification still had significantly poor survival compared with the patients without *AIB1* amplification **D2.**

To determine the role of AIB1 in gastric tumorigenesis, we first examined mRNA levels of *AIB1* in 30 pairs of primary gastric cancer tissues and matched normal gastric tissues by using quantitative RT-PCR (qRT-PCR) assay. As shown in Fig. 1A, compared with matched normal gastric tissues, *AIB1* was up-regulated in 21 of 30 (70.0%) gastric cancer tissues (*P* = 0.0002). Given that genomic amplification is one of the major causes of oncogene overexpression in human cancers including gastric cancer [9, 10], we analyzed the copy number of *AIB1* gene in 133 paraffin-embedded gastric cancers and 37 control subjects by using real-time quantitative PCR method. Copy number of *AIB1* gene corresponding to each individual case was shown in Fig. 1B1. Further analysis indicated that copy number of *AIB1* gene in gastric cancer tissues was significantly higher than control subjects (*P* < 0.0001). With a gene copy number of 4 or more defined as gene amplification, *AIB1* amplification was found in 47 of 133 (35.3%) gastric cancers, but not in control subjects. Some of the data were also confirmed by using fluorescence in situ hybridization (FISH) in primary gastric cancers (Fig. 1B2).

To explore the relationship between of copy number of *AIB1* and its protein expression, we randomly selected 12 paraffin-embedded gastric cancer cases with different *AIB1* copies and did immunohistostaining for AIB1. As shown in Fig. 1C1, increased staining of AIB1 was seen with increased *AIB1* copies. Linear regression analysis on the 12 cases revealed a positive correlation between AIB1 immunohistostaining score and *AIB1* copies (Fig. 1C2; *r* = 0.87). Similarly, we also found a close association of mRNA expression levels of *AIB1* with its copy number in 30 paired primary gastric cancer cases. As shown in Fig. 1C3, there was a significantly positive relationship between *AIB1* overexpression and its genomic amplification (*P* = 0.022). However, mRNA levels of *AIB1* were also higher in the cases without *AIB1* amplification than matched normal gastric tissues (*P* = 0.012), indicating the existence of other possible mechanisms leading to its overexpression.

### Association of *AIB1* amplification with poor prognosis in gastric cancer

Given frequent *AIB1* amplification in gastric cancers, but not in normal gastric tissues, we investigated the association of *AIB1* amplification with clinical outcomes in a cohort of gastric cancers. As shown in Table 1, *AIB1* amplification was significantly associated with age (*P* = 0.003), differentiation (*P* = 0.03), tumor invasion (*P* = 0.04) and survival status (*P* = 0.006). Although no statistical significance was noted, there was a positive association of *AIB1* amplification with TNM stage (*P* = 0.08). To evaluate the independent association of *AIB1* amplification with gender, age, differentiation, TNM stage and survival status, we further conducted a multiple multivariable logistic regression (Table 2). Similarly, after adjustment, *AIB1* amplification was still closely associated with age (OR = 1.90, 95% CI = 1.27-2.84; *P* = 0.002), differentiation (OR = 3.23, CI = 1.38-7.55; *P* = 0.007) and survival status (OR = 2.23, 95% CI = 0.89-5.61; *P* = 0.08).

**Table 1:** Association of *AIB1* amplification with clinicopathologic characteristics in gastric cancers

Variables	*AIB1* amplification (*n* = 133)		
	Yes	No	*P*
No. of patients	47	86	
Gender			
Male	67	37	0.91
Female	19	10	
Age, years			
Mean ± SD	62.68 ±13.83	57.38 ±12.33	0.003
≤ 50	9	25	
50–60	4	26	
60–70	21	25	
> 70	13	10	
Tumor localization			
gastric cardia	16	20	0.41
gastric body	10	25	
gastric antrum	21	41	
Tumor size (cm^3^)			
≤ 3	10	33	0.12
3–5	20	27	
> 5	17	26	
Differentiation			
well/moderate	14	43	0.03
poor/undifferentiation	33	43	
Tumor invasion			
T1/T2	8	29	0.04
T3/T4	39	57	
TNM stage			
I	6	25	0.08
II	8	13	
III	31	44	
IV	2	4	
Lymph node metastasis			
Yes	32	49	0.21
No	15	37	
Survival status			
Dead	31	35	0.006
Alive	16	51	

**Table 2:** *AIB1* amplification in gastric cancers — multivariable models assessing selected clinicopathologic characteristics

Characteristics	OR^†^(95% CI)	*P*
Gender	0.95 (0.39–2.46)	0.95
Age^1^	1.90 (1.27–2.84)	0.002
Differentiation^2^	3.23 (1.38–7.55)	0.007
TNM stage^3^	0.97 (0.57–1.65)	0.92
Survival status^4^	2.23 (0.89–5.61)	0.08

† OR: odds ratio with 95% confidence interval

1 Age (per 10 years)

2 Differentiation (well or moderate; poor or no differentiation)

3 TNM stage (I; II; III; IV)

4 Survival status (Alive vs. Dead)

To determine the effect of *AIB1* amplification on patient survival, univariate cox regression analysis was performed in this study. As shown in Table 3, *AIB1* amplification was significantly associated with poor survival with a hazard ratio (HR) of 2.23 (95% CI = 1.37-3.62; *P* = 0.001). Next, Kaplan-Meier method was used to evaluate the impact of *AIB1* amplification on patient survival. As shown in Fig. 1D1, the patients with *AIB1* amplification had significantly shorter median survival times than the patients without *AIB1* amplification (36.2 months *vs*. 57.5 months; *P* = 0.001). Increasing evidences have shown that residual tumor after surgery is an independent risk factor for gastric cancer patients [9]. Thus, we excluded the patients with residual tumor to test the effect of *AIB1* amplification on patient survival. Similarly, *AIB1* amplification still significantly shortened median survival times of gastric cancer patients (38.9 months *vs*. 58.8 months, *P* = 0.006) (Fig. 1D2). To further assess the prognostic value of *AIB1* amplification in gastric cancer, multivariate Cox regression analysis was performed in this study. The results indicated that *AIB1* amplification might be served as a predictor of poor survival in gastric cancer (HR = 1.85; 95% CI = 1.08–3.18; *P* = 0.03) as an independently variable with respect to gender, age, differentiation and TNM stage (Table 3).

**Table 3:** Prognostic values of *AIB1* amplification in univariate and multivariate Cox regression analysis (*n* = 133)

Characteristics	Univariate analysis		Multivariate analysis	
	HR^†^ (95% CI)	*P*	HR^†^ (95% CI)	*P*
*AIB1* amplification	2.23 (1.37–3.62)	0.001	1.85 (1.08–3.18)	0.03
Gender	0.91 (0.50–1.67)	0.76	0.77 (0.42–1.49)	0.42
Age^1^	1.35 (1.07–1.72)	0.01	1.09 (0.84–1.45)	0.41
Differentiation^2^	1.45 (0.88–2.39)	0.14	1.28 (0.76–2.15)	0.36
TNM stage^3^	2.81 (1.94–4.08)	<0.0001	1.66 (1.00–2.75)	0.08

† HR: Hazard Ratio

1 Age (per 10 years)

2 Differentiation (well or moderate; poor or no differentiation)

3 TNM stage (I; II; III; IV)

### AIB1 down-regulation inhibits gastric cancer cell growth

**Figure 2:**
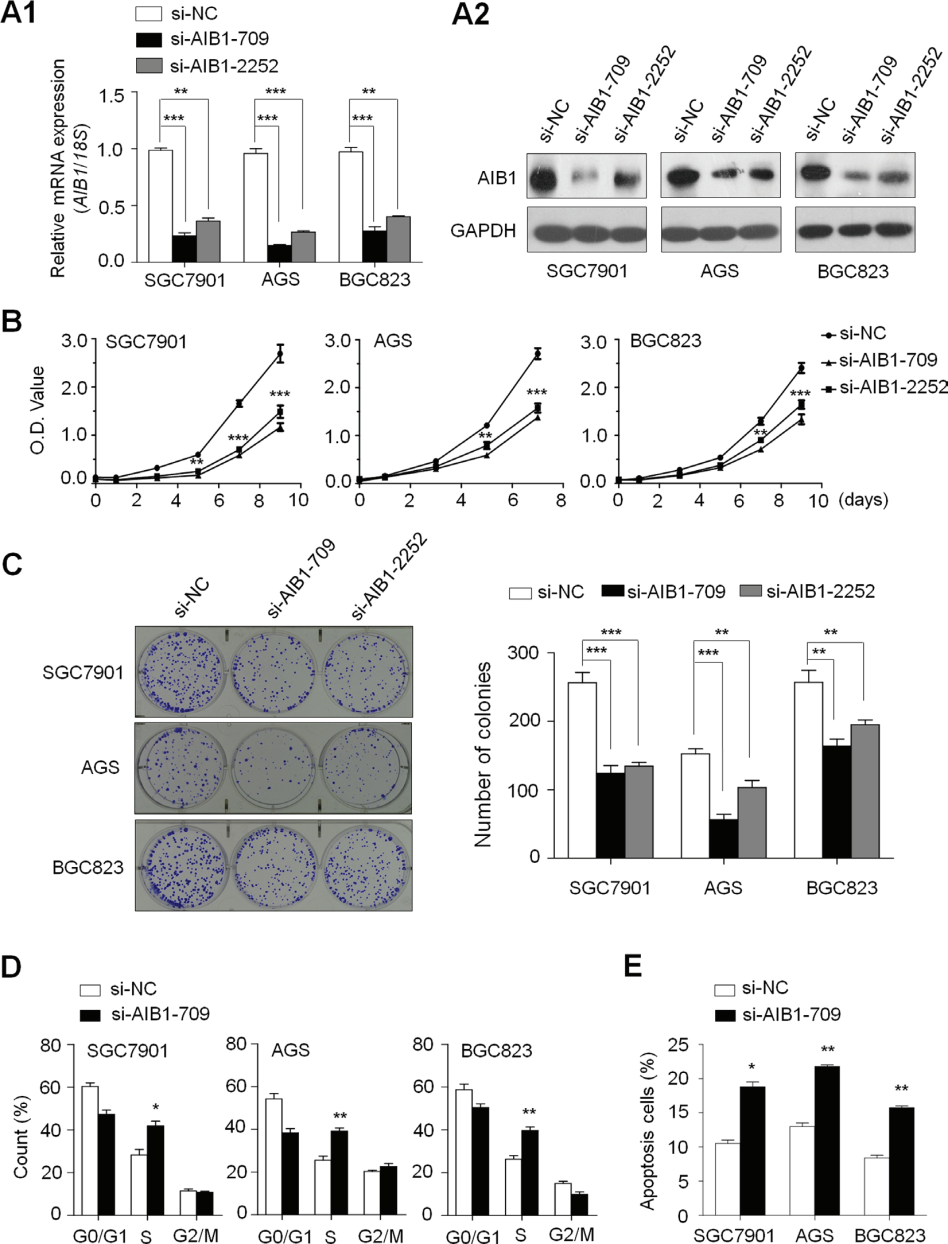
Inhibition of cell growth and induction of cell cycle arrest and apoptosis by AIB1 knockdown in gastric cancer cells Knockdown of *AIB1* mRNA **A1.** and protein **A2.** by using two different siRNAs (si-AIB1-709 and si-AIB1-2252) in gastric cancer cell lines SGC7901, AGS and BGC823 was evidenced by qRT-PCR and western blot, respectively. *18S* rRNA was used as a normalized control for qRT-PCR assay. GAPDH was used as loading control in western blot analysis. ***P < 0.001. **B.** AIB1 down-regulation significantly inhibited cell proliferation in gastric cancer cells. *P < 0.05; **P < 0.01; ***P < 0.001. **C.** The effect of AIB1 knockdown on cell growth was further confirmed by colony formation assay. Left panel shows the representative images of colony formation in cells transfected with si-AIB1s or si-NC. Quantitative analysis of colony numbers is shown in right panel. Data were presented as mean ± SE of values from three different assays. **P < 0.01; ***P < 0.001. **D.** Cells were transiently transfected with si-AIB1-709 or si-NC. DNA content was measured by flow cytometry to determine cell cycle fractions. The fraction of cells in each cell cycle phase was indicated in the figures. Data were presented as mean ± SE of values from three independent experiments. *P < 0.05; **P < 0.01. **E.** Cells transiently transfected with si-AIB1-709 or si-NC. Apoptotic cells including early and late apoptotic cells were measured 72 hours after transfection by flow cytometry analysis of Annexin V-FITC/PI double-labelled cells. The experiment was repeated three times and data were presented as mean ± SE. **P < 0.01.

Frequent overexpression and amplification of *AIB1* in primary gastric cancers but not in control subjects suggests that *AIB1* may play an oncogenic role in gastric tumorigenesis. Thus, we tested the growth-suppressive effect through down-regulating *AIB1* expression in gastric cancer cell lines SGC7901, AGS and BGC823 with *AIB1* overexpression but not genomic amplification. AIB1 down-regulation by two different siRNAs (si-AIB1-709 and si-AIB1-2252) was confirmed by qRT-PCR and western blot assays (Fig. 2A). These two specific *AIB1* siRNAs significantly inhibited cell proliferation as compared with control siRNA (si-NC) (Fig. 2B). The inhibitory effect on gastric cancer cell growth was further confirmed by colony formation assay. As shown in Fig. 2C, down-regulating AIB1 expression in these cells significantly decreased colony forming ability in monolayer culture. Taken together, these findings suggest that AIB1 plays a growth-promoting activity in gastric cancer.

### AIB1 down-regulation induces cell cycle arrest and apoptosis in gastric cancer cells

We also tested the contribution of cell cycle arrest and apoptosis to the observed growth-inhibitory of cells transfected with si-AIB1-709. As shown in Fig. 2D, cell cycle was arrested at the S phage in si-AIB1 transfected cells as compared with si-NC transfected cells. The percentage of S phase was increased from 28.2 ± 4.5% to 41.9 ± 3.7% in SGC7901 cells (*P* = 0.02), from 25.5 ± 3.4% to 39.1 ± 2.5% in AGS cells (*P* = 0.005) and from 26.3 ± 2.8% to 39.7 ± 3.0% in BGC823 cells (*P* = 0.005), respectively. Next, we investigated the impact of AIB1 down-regulation on cell apoptosis. As shown in Fig. 2E, si-AIB1 transfection showed an increase in cell apoptosis as compared with control cells (18.8 ± 0.9% *vs*. 10.5 ± 0.6% in SGC7901 cells, *P* = 0.01; 21.8 ± 0.4% *vs*. 13.0 ± 0.7% in AGS cells, *P* = 0.004; 15.8 ± 0.4% *vs*. 8.4 ± 0.6% in BGC823 cells, *P* = 0.005).

### Association of AIB1 down-regulation with xenograft tumor growth

**Figure 3:**
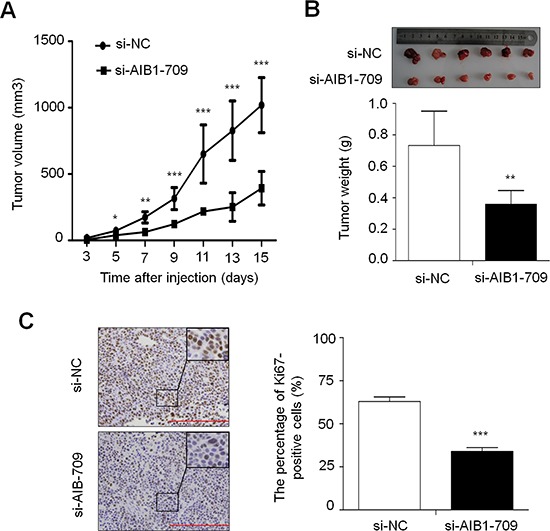
Effect of AIB1 down-regulation on xenograft tumor growth **A.** Subcutaneous tumor growth curve of si-AIB1-709 transfected cells in nude mice was compared with si-NC transfected cells. The si-AIB1-709 group showed a retarded tumor growth compared to the si-NC group. Data were presented as mean ± SD (*n* = 6/group). *P < 0.05; **P < 0.01; ***P < 0.001. **B.** A representative picture for tumor growth of cells transfected with the indicated siRNA in nude mice (upper panel). Histogram represents mean of tumor weight from the si-AIB1-709 and si-NC groups (lower panel). Data were presented as mean ± SD (*n* = 6/group). **P < 0.01. **C.** Shown is representative Ki-67 staining of xenograft tumors from the si-AIB1-709 and si-NC groups (left panel). Histogram represents mean ± SE of the percentage of Ki-67-positive cells from 5 microscopic fields in each group (right panel). ***P < 0.001.

Given inhibitory effect of AIB1 knockdown on gastric cancer cell growth *in vitro*, we thus assessed the effect of AIB1 down-regulation on the growth of xenograft tumors in nude mice. As shown in Fig. 3A, compared with si-NC transfected cell-derived xenograft tumors, si-AIB1 transfected cell-derived xenograft tumors grew more slowly. The volume of si-AIB1 transfected cell-derived xenograft tumors was significantly lower than si-NC transfected cell-derived xenograft tumors. At the end of experiments, tumors were isolated and weighed. The mean weight of si-AIB1 transfected cell-derived xenograft tumors was significantly less as compared with si-NC transfected cell-derived xenograft tumors (*P* = 0.003) (Fig. 3B). To quantitatively assess the proliferation index in xenograft tumors, tumor sections were stained for Ki-67 expression. As shown in Fig. 3C, si-AIB1 significantly decreased the percentage of Ki-67 positive cells in tumors as compared with si-NC (*P* < 0.001). These observations further support the growth-promoting effect of AIB1 in gastric cancer.

### AIB1 down-regulation inhibits gastric cancer cell migration and invasion

**Figure 4:**
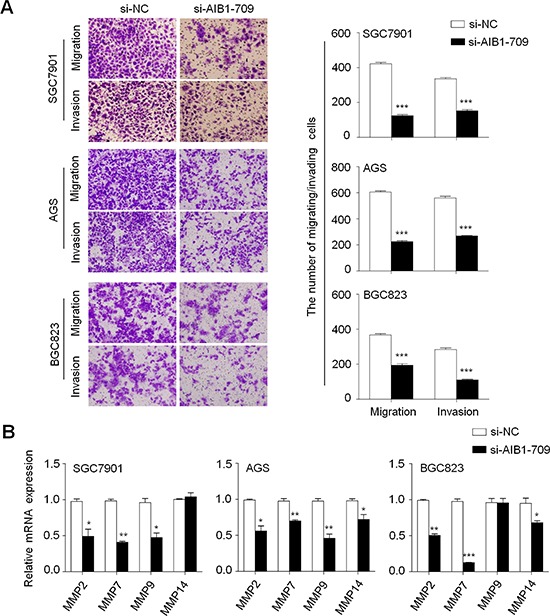
Inhibition of gastric cancer cell migration and invasion by AIB1 down-regulation **A.** Cells transfected with si-AIB1-709 or si-NC were starved overnight and then seeded in the transwell chambers without matrigel for migration assay, and coated with matrigel for invasion assay, respectively. After a 24 h-culture, non-migrating (or non-invading) cells in the upper chamber were removed and migrating (or invading) cells were stained and calculated in five microscopic fields per sample. Shown are representative images of migrating (or invading) cells (left panels). Histograms (right panels), corresponding to left panels, show means ± SE of the numbers of migrating (or invading) cells from three independent assays. ***P < 0.001. **B.** qRT-PCR assay was performed to investigate the effect of *AIB1* knockdown on the expression of metastasis-related genes *MMP-2*, *-7*, *-9* and *-14* in gastric cancer cells. Expression levels of these genes were normalized with *18S* rRNA levels. Data were presented as mean ± SE. *P < 0.05; **P < 0.01; ***P < 0.001.

Given that metastasis is the main cause of cancer-related death and AIB1 has been demonstrated to be involved in cancer metastasis [15, 28, 29], we thus attempted to investigate the effect of AIB1 down-regulation on the migration and invasion abilities of gastric cancer cells in this study. As shown in Fig. 4A, there were a significantly lower number of migrated cells in si-AIB1 transfected cells than in si-NC transfected cells. Moreover, the invasion assay showed that AIB1 knockdown significantly decreased the ability of cells to pass through the Matrigel-coated membrane. These results suggest that AIB1 knockdown significantly inhibits the migration and invasive potential of gastric cancer cells. Given a critical role of matrix metalloproteinases (MMPs) in cell migration and invasion [30], we next tested the effect of AIB1 down-regulation on the expression of *MMP-2*, *-7*, *-9* and *-14 g*enes in gastric cancer cells. As expected, AIB1 knockdown significantly inhibited the expression of these genes in at least two cell lines (Fig. 4B), suggesting that decrease in the metastasis-associated phenotypes may be link to the inhibition of MMPs in gastric cancer.

**Figure 5:**
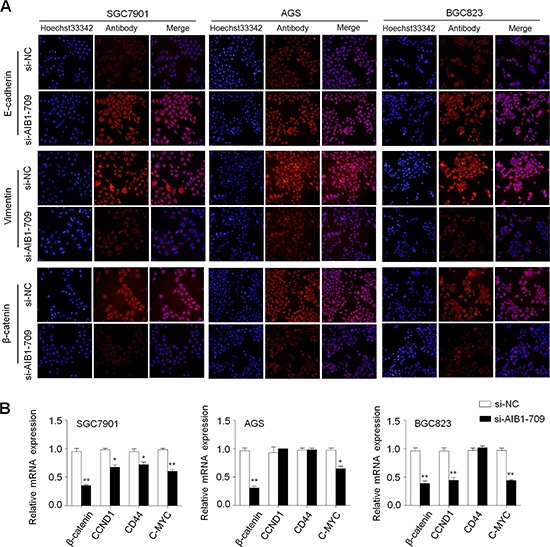
Effect of AIB1 down-regulation on the process of EMT and the expression of β-catenin and its target genes in gastric cancer cells **A.** Cells transfected with si-AIB1-709 or si-NC were seeded on the coverslips in 6-well plates. After a 48 h-culture, immunofluorescence staining was then performed to assess the expression of E-cadherin, Vimentin and β-catenin proteins in SGC7901, AGS and BGC823 cells. Red color represents target protein fluorescence and blue color represents Hoechst33342 staining for nuclei. **B.** qRT-PCR assay was performed to assess the effect of AIB1 knockdown on the expression of *β-catenin* and its target genes in gastric cancer cells. Expression levels of these genes were normalized with 18S rRNA levels. Data were presented as mean ± SE. *P < 0.05; **P < 0.01.

To further explore the mechanism of AIB1 contributing to cell migration and invasion, we investigated its effect on the process of epithelial-mesenchymal transition (EMT), which is one of the critical steps during tumor metastasis including gastric cancer [31]. As shown in Fig. 5A, immunofluorescence assay indicated that knocking down *AIB1* expression in gastric cancer cells substantially increased the expression of epithelial cell marker E-cadherin and reduced the expression of mesenchymal marker vimentin. These results indicate that suppression of the EMT process by AIB1 down-regulation may contribute to inhibition of gastric cancer cell migration and invasion.

It is well documented that β-catenin is an integral structural component of cadherin-based adherens junctions, and the key nuclear effector of canonical Wnt signaling. Its imbalance in the structural and signaling properties can cause disease and deregulated growth connected to cancer and metastasis [32]. Thus, we also tested the effect of AIB1 down-regulation on the expression of β-catenin and its target genes *CCND1* (also known as *cyclinD1*), *CD44* and *C-MYC* in these cells. As expected, AIB1 knockdown significantly decreased the expression of β-catenin protein and mRNA in these cells (Fig. 5), as well as reduced the expression of its target genes in at least one cell lines (Fig. 5B). These observations suggest the involvement of AIB1 in the regulation of Wnt/β-catenin pathway, further contributing to gastric cancer cell growth and metastasis.

### AIB1 modulates major signaling pathways in gastric cancer

**Figure 6:**
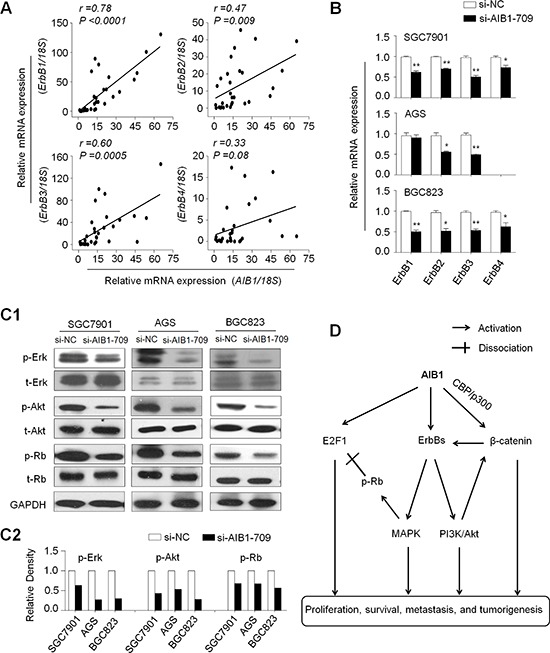
Modulation of major signaling pathways by AIB1 **A.** qRT-PCR assay was used to evaluate the mRNA expression of *AIB1* and ErbB receptors in primary gastric cancers (*n* = 30). Linear regression analysis was performed to assess the correlations between them. *18S* rRNA was used as a normalized control. **B.** qRT-PCR assay was performed to test the effect of AIB1 knockdown on the expression of ErbB receptors in gastric cancer cells. *18S* rRNA was also used as a normalized control. *P < 0.05;**P < 0.01. **C1.** Cells transfected with si-AIB1-709 or si-NC were lysed and lysates were subjected to western blot assays. The antibodies against phospho-Erk (p-Erk), total Erk (t-Erk), phospho-Akt (p-Akt) and total Akt (t-Akt) were used to determine the effect of AIB1 down-regulation on the activities of the MAPK and PI3K/Akt pathways. The antibodies against Rb and phosphorylated Rb (p-Rb) were used to test the effect of AIB1 down-regulation on Rb signaling. GAPDH was used as a loading control. **C2.** Shown was quantitative illustration of levels of p-Erk, p-Akt, and p-Rb using densitometry to measure the density of the corresponding bands on Western blot shown in (C1). **D.** Schematic model of molecular mechanisms underlying oncogenic role of AIB1 in gastric cancer. *AIB1* overexpression or amplification can up-regulate the expression of ErbB receptors and subsequently activates ErbB-mediated signalling pathways such as MAPK and PI3K/Akt pathways. AIB1 also modulates Rb/E2F1 signaling through promoting phosphorylation of Rb and co-activating E2F1 transcriptional activity. In addition, the previous evidences and the findings in the present study demonstrate that AIB1 can activate Wnt/β-catenin signaling through different mechanisms. Taken together, *AIB1* overexpression or amplification promotes gastric cancer cell growth and invasiveness through modulating major signaling pathways, ultimately contributing to poor prognosis of gastric cancer patients.

Given that ErbB receptors, including ErbB1, ErbB2, ErbB3 and ErbB4 (also known as HER1, HER2, HER3 and HER4), are a group of receptor tyrosine kinases (RTKs) involved in key cellular functions such as cell growth, survival and metastasis during tumorigenesis including gastric cancer [33], we thus want to determine whether oncogenic role of AIB1 in gastric cancer is associated with the activation of ErbB receptors. As shown in Fig. 6A, *AIB1* expression was strongly positively correlated with the expression of *ErbB1*, *ErbB2*, *ErbB3* and *ErbB4*, particularly the first three genes. Accordingly, *AIB1* knockdown dramatically decreased the expression of ErbB receptors in at least two cell lines as compared with controls (Fig. 6B).

Accumulated evidences have demonstrated that overexpression of ErbB receptors leads to the activation of downstream signaling pathways including Ras/MAPK and phosphatidylinositol-3-kinase (PI3K)/Akt pathways [33–35]. Next, we investigated the effect of AIB1 on the activities of these two pathways by western blot analysis. As shown in Fig. 6C, AIB1 down-regulation significantly inhibited the activities of both pathways in gastric cancer cells, characterized by reduced phosphorylation of Erk (p-Erk) and Akt (p-Akt). It is clear that the retinoblastoma protein Rb is critical for the regulation of mammalian cell cycle entry [36], and a previous study has demonstrated that Erk1/2 cascade as the first MAPK pathway elucidated [37] is responsible for Rb phosphorylation [38]. We thus speculate that AIB1 may regulate the activity of Rb/E2F signaling in gastric cancer. As expected, our data showed that the expression of phosphorylated Rb (p-Rb) was significantly decreased in si-AIB1 transfected cells as compared with si-NC transfected cells (Fig. 6C). Collectively, these findings suggest that *AIB1* functions as a critical oncogene in gastric cancer through modulating major signaling pathways.

## DICUSSION

In this study, we discovered four lines of evidence supporting the potential oncogenic activities of AIB1 in gastric carcinogenesis. First, *AIB1* was frequently overexpressed and amplified in gastric cancer tissues as compared with control subjects. Second, *AIB1* amplification was strongly associated with poor survival and may be used as a potential prognostic marker for gastric cancer patients. Third, knocking down *AIB1* expression in gastric cancer cells significantly inhibited cell growth and invasiveness. Fourth, AIB1 may modulate major signaling pathways such as ErbB and Wnt/β-catenin signaling pathways in gastric cancer.

*AIB1* has been found to be overexpressed or amplified in multiple human cancers including gastric cancer and played an important role in tumorigenesis [19, 27, 39]. To elucidate the association of *AIB1* amplification with its expression, we examined copy number of *AIB1* and its mRNA/protein expression in a cohort of primary gastric cancers, and demonstrated a significantly positive relationship between them. However, similar to the findings in a previous study [27], our data showed that *AIB1* overexpression did not always coincide with its genomic amplification, suggesting that *AIB1* expression is likely regulated not only by gene amplification but also by other mechanisms such as transcriptional activation or miR-17–5p [40]. In addition, our data demonstrated that *AIB1* amplification was associated with a significantly increased risk of cancer-related death and dramatically affected the survival of gastric cancer patients, which was consistent with a previous study [27]. Collectively, these data suggest that *AIB1* amplification would be one of the major mechanisms of *AIB1* overexpression in gastric cancer and may be used as a potential prognostic marker for this disease.

Although a previous study has demonstrated *AIB1* amplification and overexpression in gastric cancer [27], the role of AIB1 in gastric tumorigenesis and dysregulation of signaling pathways mediated by AIB1 remains totally unknown. Thus, we tested the oncogenic effect of AIB1 in gastric cancer by using both *in vitro* and *in vivo* assays. AIB1 knockdown in gastric cancer cells showed significant growth-suppressing effect by inhibition of cell proliferation and colony formation *in vitro* and tumorigenic potential in nude mice. Moreover, AIB1 down-regulation induced cell cycle arrest and apoptosis and inhibited cell migration, invasion and EMT process in gastric cancer cells. We also demonstrated that AIB1 down-regulation significantly inhibited expression of *MMP-2*, *-7*, *-9* and *-14 g*enes in gastric cancer cells, suggesting that the decrease in the metastasis-associated phenotypes may be mediated by suppressing expression or activities of MMPs. These findings further confirm that *AIB1* is a critical oncogene in gastric cancer.

To better understand oncogenic role of AIB1 in gastric tumorigenesis, we tested its effect on major signaling pathways in gastric cancer cells. A previous study revealed a positive correlation between the expression of *AIB1* and ErbB receptors, contributing to an early response to endocrine treatment in breast cancer [41]. Another study showed that AIB1 knockdown reduced EGF-mediated phosphorylation of *ErbB1* and *ErbB2*, and proposed a portion of the oncogenic effect of *AIB1* could be through regulation of *ErbB1* and *ErbB2* activity and subsequent modulation of downstream cellular signaling pathways [42]. These findings were supported by the present study that there were strongly positive correlations between the expression of *AIB1* and ErbB receptors in gastric cancers, and knocking down *AIB1* expression in gastric cancer cells significantly reduced the expression of ErbB receptors. As expected, our data indicated that AIB1 down-regulation remarkably inhibited the activity of ErbB-mediated signaling pathways such as the MAPK and PI3K/Akt pathways. To be consistent with this, several studies have demonstrated that AIB1 promotes tumor progression *in vitro* and *in vivo* through activating the PI3K/Akt pathway [13, 20, 25]. Notably, we found that AIB1 down-regulation significantly decreased the levels of p-Rb through inhibiting the activity of MAPK pathway. It is well known that, once Rb is phosphorylated, it is no longer capable of binding E2F transcription factor such as E2F1 and triggers the transcription of E2F1 targeted genes [36]. In addition, a previous study has demonstrated that AIB1 acts as an E2F1 coactivator and is required for E2F1-mediated transcriptional activation of gene expression [12]. These observations suggest that AIB1 contributes to cell proliferation partially through modulating Rb/E2F1 signaling pathway.

The Wnt/β-catenin pathway plays a crucial role in tumorigenesis [32, 43]. In this study, knocking down *AIB1* expression in gastric cancer cells significantly reduced the expression of β-catenin and its target genes, as supported by two previous studies that AIB1 directly interacted with the general transcriptional cointegrator CBP/p300 through CBP-interaction domain (CID) in the C-terminal region [44], and interaction of CBP with β-catenin can activate gene expression [45]. In addition, β-catenin may form heterodimer with ErbB1/EGFR to activate ErbB1 pathway, and ErbB1 can also transactivate β-catenin through receptor tyrosine kinase-PI3K/Akt pathway [46]. These observations suggest that Wnt/β-catenin signaling contributes to oncogenic effect of AIB1 in gastric tumorigenesis.

In summary, we found frequent overexpression and amplification of *AIB1* in gastric cancer and demonstrated a strong association of *AIB1* amplification with poor patient survival. Our data are consistent with a model (Fig. 6D) in which AIB1 contributes to gastric carcinogenesis by promoting cell growth and invasiveness through modulating a broad spectrum of signal pathways. Given the complexity of the signaling network that AIB1 participates in and regulates, it may represent a potential therapeutic target for gastric cancer. Thus, targeting AIB1 with specific inhibitors holds future promise for clinical cancer therapy [47]. Until now, two small molecular drugs gossypol and buffalo have been found as AIB1 inhibitors [48, 49]. However, they are not specific for AIB1. Extensive further study will be required to search for improved inhibitors and take these approaches beyond the preclinical setting.

## MATERIALS AND METHODS

### Tissue samples

With the approval of institutional review board and human ethics committee, a total of 30 matched pairs of resected primary gastric cancers and normal gastric tissues were obtained from the First Affiliated Hospital of Xi'an Jiaotong University School of Medicine. In addition, formalin-fixed and paraffin-embedded tissue sections from 133 gastric cancer patients were randomly obtained from the First Affiliated Hospital of Xi'an Jiaotong University School of Medicine between January 1999 and December 2005. The normal controls from 37 patients with chronic gastritis who underwent endoscopic biopsy, were also obtained from the same hospital. Informed consent was obtained from each patient before the surgery. All patients did not receive chemotherapy and radiotherapy before the surgery, and all sections were histologically examined by a senior pathologist at Department of Pathology of the Hospital based on World Health Organization (WHO) criteria. Clinicopathological data were obtained from the patients' files or by interview with the patients or their relatives, and were summarized in Table 1.

### Cell culture and short interfering RNA (siRNA) transfection

Human gastric cancer cell lines AGS, BGC823 and SGC7901 were cultured in RPMI 1640 medium supplemented with 10% fetal bovine serum (FBS). The oligonucleotides of siRNA targeting AIB1 (si-AIB1-709 and si-AIB1-2252) and control siRNA (si-NC) were obtained from GenePharma (Shanghai, P.R. China) and the sequences were presented in Supplementary Table S1. Cells were transfected at 70% confluence using Lipofectamine 2000 (Invitrogen, Grand Island, NY), with a final siRNA concentration of 50 nM. Specific oligonucleotides with maximal knockdown efficiency were selected among three different sequences until use.

### RNA extraction and quantitative RT-PCR (qRT-PCR)

Total RNA from tissues and cell lines were isolated by TRIzol reagent following manufacturer's instruction (Takara Inc., Dalian, P.R. China), and cDNA was prepared using PrimeScript RT reagent Kit (Takara Inc., Dalian, P.R. China). Quantitative RT-PCR (qRT-PCR) was carried out on a CFX96 Thermal Cycler Dice™ real-time PCR system (Bio-Rad Laboratories, Inc., CA) using SYBR Premix Ex Taq™ (Takara Inc., Dalian, P.R. China). The mRNA expression of the indicated genes was normalized to 18S rRNA cDNA. Each sample was run in triplicate. The primer sequences were presented in Supplementary Table S2.

### Tissues and DNA preparation

Paraffin-embedded serial sections were made at intervals of 5 μm. One of sections was stained by hematoxylin and eosin (H&E) and was marked as a tumor representative tissue by a senior pathologist for gastric cancer. Tumor tissues were then isolated by manual microdissection under an inverted microscope using the marked H&E section as target tissue identification. DNA was extracted from isolated tissues as previously described [9]. Briefly, after a treatment for overnight at room temperature with xylene to remove pareffin, tissues were digested with 1% sodium dodecyl sulfate (SDS) and 0.5 mg/ml proteinase K at 48°C for 48 h, with addition of several spiking aliquots of concentrated proteinase K to facilitate digestion. DNA was subsequently isolated using standard phenol/chloroform protocol. Subsequent sections were mounted on 3-aminopropyltriethoxysilane-coated slides for immunohistochemical assay.

### Copy number analysis

Real-time quantitative PCR approach was performed to analyze the copy number of AIB1 gene in primary gastric cancers and control subjects on a CFX384 Thermal Cycler Dice™ real-time PCR system (Bio-Rad Laboratories, Inc., CA) as described previously [9,10]. Specific primers and TaqMan probes were designed using Primer Express 3.0 (Applied Biosystems) to amplify both the *AIB1* and internal reference gene *β-actin*. For the *AIB1* gene, the TaqMan probe used was 5′-6FAM-ATC TGT GTG GCA CGC CGC ATT ACT ACA-TAMRA-3′, and the primers were 5′-CCT TAC CAG GGT GAA TTT TTT ATT G-3′ (forward) and 5′-GGG TTT GAT GGA AAT GTT CTT TCT-3′ (reverse). The TaqMan probe and primers for *β-actin* gene were described previously [9, 10]. Using a PCR protocol described previously [9, 10], each sample was run in triplicate, and *β-actin* gene was run in parallel to normalize input DNA. Standard curves were established using serial dilutions of normal leukocyte DNA with a quantity range of 3.75-60 ng per 2μL. *AIB1* amplification o was defined by a copy number ≥ 4.

### Fluorescence in situ hybridization (FISH)

Two-color FISH was performed on formalin-fixed, paraffin-embedded gastric cancer tissues using the AIB1 DNA probe/CEN20 probe mixture (Exon Biotechnology Inc, Guangzhou, China). Briefly, the paraffin-embedded tissue slides were deparaffinized through xylene, and rehydrated in an ethanol series (100%, 85% and 70%, and treated with pepsin at 37°C, respectively. The slides were then dehydrated in an ethanol series (70%, 85% and 100%), and the probe mixture was applied to the slides and immediately covered by coverslips and sealed the edges with rubber cement. Subsequently, the slides were denatured in the hybrid apparatus at 85°C for 5 minutes and incubated at 37°C overnight. After hybridization, the slides were washed in 2 × SSC, 2 × SSC/0.1% NP-40 buffer at 37°C for 5 minutes each, and were counterstained with DAPI antifade solution. FISH signals in 20-30 cells for each specimen were counted, and the criteria for gene amplification were defined when FISH signals were detected by tested probes compared with control probes ≥ 1.5. Fluorescence images were captured with Olympus IX71 microscope (Olympus, Tokyo, Japan), which enables simultaneous detection of both FITC and Texas Red fluorescence. The color mergence was performed using ImageJ image software (ImageJ version 1.44p, NIH, MD).

### Immunohistochemistry (IHC)

Immunohistochemistry (IHC) was performed to investigate the relationship between the expression levels of AIB1 protein and copy number of *AIB1* gene in the tumor tissues as described previously [9]. Briefly, paraffin-embedded tissue sections (5 μm) were deparaffinized and rehydrated in xylene and degradation alcohol. After antigen retrieval using microwave heating for 15 min, the slides were washed and incubated with anti-AIB1 antibody (Abcam, Inc) overnight at 4°C. Immunodetection was performed with the Streptavidin-Peroxidase system (ZSGB-bio, Beijing, China) according the manufacture's protocol, followed by reaction with diaminobenzidine and counterstaining with hematoxylin. To insure the comparability of immunohistochemical staining, a common reference standard was included as an internal or intra-assay control in each batch. AIB1 protein expression was scored in double-blinding way (i.e., without knowing the *AIB1* copy number of the case), and 0, 1, 2, 3 reprents negative, weak positive, positive, and strong positive, respectively.

### Western blot analysis

Cells were lysed in prechilled RIPA buffer containing protease inhibitors. Equal amounts of protein lysates were separated by SDS–PAGE and transferred onto PVDF membranes (Roche Diagnostics, Mannheim, Germany). The membranes were then incubated with primary antibodies. Anti-AIB1 and anti-total-Erk1/2 (t-Erk) were purchased from Abcam, Inc. Anti-phospho-Akt^Ser473^, anti-phospho-Erk1/2 and anti-total-Akt (t-Akt) were purchased from Bioworld Technology, co, Ltd. Anti-total-Rb (t-Rb) and anti-phospho-Rb^S811^ (p-Rb) were purchased from Epitomics, Inc. Anti-GAPDH was purchased from Abgent, Inc. This was followed by incubation with species-specific HRP-conjugated secondary antibodies from ZSGB-BIO, and immunoblotting signals were visualized using the Western Bright ECL detection system (Advansta, CA).

### Cell proliferation and colony formation assays

Cell proliferation was measured every 2 days by the MTT assay. Briefly, cells transfected with si-AIB1-709 and -2252 or si-NC (1000/well) were seeded and cultured in 96-well plates. At the indicated times, 20 μl of 0.5 mg/ml MTT (Sigma, Saint Louis, MO) was added into the medium and the plates were further incubated for 4 h, followed by adding 150 μl of DMSO. The plates were then read on a microplate reader using a test wavelength of 570 nm and a reference wavelength of 670 nm. All MTT assays were done in triplicate.

Colony formation assay was performed using monolayer culture. Cells (800/well) transfected with different siRNAs were cultured in 6-well plates. The medium was refreshed every 3 days. After 14 days of culture, surviving colonies (≥ 50 cells per colony) were fixed with methanol and stained with 0.5% crystal violet, and the colonies were then counted. Each experiment was performed in triplicate.

### Cell cycle and apoptosis assays

For cell cycle analysis, 2 day after siRNA transfection, cells were synchronized by serum starvation for 12 h and induced to reenter the cell cycle by an exchange of 10% FBS. Following this, cells were harvested at 24, 48, and 72 h after replacing the medium and fixed in ice-cold 70% ethanol at 4°C overnight. Cells were then stained with propidium iodide solution (50 μg/mL propidium iodide, 50 μg/mL RNase A,0.1% Triton-X, 0.1 mM EDTA), and subjected to FACS analysis (BD Biosciences, NJ).

For apoptosis analysis, the indicated cells were harvested, washed with PBS, suspended in binding buffer, and sequentially stained with Annexin V-FITC Detection Kit (Roche Applied Science, Penzberg, Germany) by flow cytometer according to the manufacturer's protocol. Each experiment was performed in triplicate.

### Tumor xenograft models

Male, 4- to 5-week-old nude mice were purchased from Shanghai SLAC laboratory Animal Co., Ltd. (SLAC), China. Tumor xenografts were established by subcutaneous inoculation of 4 × 10^6^ si-AIB1-709 and si-NC transfected BGC823 cells into the right armpit region of nude mice, respectively. From day 3 post-injection, tumor size was measured every 2 days. Tumor volumes were calculated by the formula (length × width^2^ × 0.5). After 15 days, tumors were harvested and weighted. Tumors obtained from representative animals were embedded in paraffin, sectioned at 4 μm, and stained with hematoxylin and eosin (H&E). Ki-67 staining was used to evaluate cell proliferation. All experimental procedures involving animals were conducted in accordance with Institution Guidelines and were approved by the Laboratory Animal Center of Xi'an Jiaotong University School of Medicine.

### Cell migration and invasion assays

Cell migration and invasion assays were assessed by transwell chambers (8.0 μm pore size; Millipore, MA). For cell invasion assay, chambers were pre-coated with Matrigel (4 × dilution; 15 μl/well; BD Bioscience, NJ). The indicated cells were starved overnight and then seeded in the upper chamber at a density of 2 × 10^4^ cells/ml in 200 μl of medium containing 0.5% FBS. Medium with 10% FBS (1 ml) was added to the lower chamber. After a 24-h incubation, non-migrating/non-invading cells in the upper chamber were removed using a cotton swab, and migrating/invading cells were then fixed in 100% methanol and stained with crystal violet solution (0.5% crystal violet in 2% ethanol). Photographs were taken randomly for five fields of each membrane. The number of migrating/invading cells was expressed as the average number of cells per microscopic field over five fields.

### Immunofluorescence staining

The indicated cells were seeded onto coverslips in 6-well plates and cultured until 70% confluence. Cells were then fixed with 4.0% formaldehyde in phosphate-buffered saline for 15 min, and blocked with 5% goat serum for 30 min. Next, the coverslips were incubated at 4°C with primary antibodies overnight. Anti-E-cadherin, anti-Vimentin and anti-β-catenin antibodies were purchased from Epitomics, Inc. Subsequently, the coverslips were incubated with Cy3-conjugated goat anti-rabbit secondary antibody (Bioss, Beijing, P.R. China) and dried, dyed with Hoechst33342, and fixed in glycerol. The images were obtained with an Olympus IX71 microscope (Olympus, Tokyo, Japan), and color mergence was performed using ImageJ image software (ImageJ version 1.44p, NIH, MD).

### Statistical analysis

The Mann-Whitney *U* test was performed to compare copy number of *AIB1* gene between gastric cancer tissues and control subjects. Association of *AIB1* amplification with clinicopathological characteristics was assessed by Fisher's exact test or Pearson's Chi square test. Multivariate models that adjusted for the most important covariates were developed by logistic regression test. Survival length was determined from the day of primary tumor surgery to the day of death or last clinical follow-up. Kaplan–Meier method was used for survival analysis grouping with *AIB1* amplification. Differences between curves were analyzed using the log-rank test. Univariate cox regression analysis was performed to determine the effect of *AIB1* amplification on patient survival. Multivariate cox regression analysis was used to evaluate the effect of *AIB1* amplification on survival of independently of gender, age, differentiation and tumor stage. All statistical analyses were performed using the SPSS statistical package (11.5, Chicago, IL). *P* values < 0.05 were considered significant.

## SUPPLEMENTAY TABLES


