# Supplementary material for: Frequent amplification of AIB1, a critical oncogene modulating major signaling pathways, is associated with poor survival in gastric cancer
Source: Oncotarget. 2015 Apr 29;6(16):14344–59. doi: 10.18632/oncotarget.3852 (PMC4546471; doi:10.18632/oncotarget.3852)
Supplement: Supplementary file 1 [file oncotarget-06-14344-s001.pdf]

## SUPPLEMENTARY TABLES

**Supplementary Table S1. The sequences of siRNAs used in this study**

| si-RNAs | Sequences (5′-3′) |
|---|---|
| si-AIB1–709 (sense) | GGACCUGGUUAACACAAGUTT |
| si-AIB1–709 (antisense) | ACUUGUGUUAACCAGGUCCTT |
| si-AIB1–2252 (sense) | GGAGGAGUAUCCUCUACAUTT |
| si-AIB1–2252 (antisense) | AUGUAGAGGAUACUCCUCCTT |
| si-NC (sense) | UUCUCCGAACGUGUCACGUTT |
| si-NC (antisense) | ACGUGACACGUUCGGAGAATT |

**Supplementary Table S2. qRT-PCR primers used in this study**

| Gene bank (ID) | Genes | Forward primer (5′-3′) | Reverse primer (5′-3′) | Product length (bp) |
|---|---|---|---|---|
| NM_001174087.1 | *AIB1* | CCGATTTAAAGCTGAGCTGC | CCAGTCAAAGGATGTTCAAGC | 111 |
| NM_001098209.1 | *β-catenin* | TCGAGGACGGTCGGACT | ATTGTCCACGCTGGATTTTC | 97 |
| NM_053056.2 | *CCND1* | GACCTTCGTTGCCCTCTGT | TGAGGCGGTAGTAGGACAGG | 140 |
| NM_000610.3 | *CD44* | GACAAGTTTTGGTGGCACG | CACGTGGAATACACCTGCAA | 105 |
| NM_002467.4 | *CMYC* | GCTGCTTAGACGCTGGATTT | CACCGAGTCGTAGTCGAGGT | 114 |
| NM_005228.3 | *ErbB1* | GGGCTCTGGAGGAAAAGAAA | AAATTCCCAAGGACCACCTC | 127 |
| NM_001005862.2 | *ErbB2* | ATCAACTGCACCCACTCCTG | TGATGAGGATCCCAAAGACCAC | 145 |
| NM_001005915.1 | *ErbB3* | AGTCATGAGGGCGAACGAC | TCACACTCAGGCCATTCAGA | 119 |
| NM_001042599.1 | *ErbB4* | ACGGGATCTGAGACTTCCAA | TTATTCTCCGTTCCTGCACA | 127 |
| NM_004530.4 | *MMP2* | TTGCTGGAGACAAATTCTGG | AAGAAGTAGCTGTGACCGCC | 148 |
| NM_002423.3 | *MMP7* | GAGCTACAGTGGGAACAGGC | GCATCTCCTTGAGTTTGGCT | 103 |
| NM_004994.2 | *MMP9* | GCACTGCAGGATGTCATAGG | ACGACGTCTTCCAGTACCGA | 128 |
| NM_004995.3 | *MMP14* | AGCCATATTGCTGTAGCCAG | GTTGTCTCCTGCTCCCCCT | 105 |
| NM_003380.3 | *Vimentin* | CTGGATTTCCTCTTCGTGGA | CGAAAACACCCTGCAATCTT | 133 |
| R_003286.2 | *18S* | CGCCGCTAGAGGTGAAATTC | CTTTCGCTCTGGTCCGTCTT | 52 |
